# Mass falls of crustacean carcasses link surface waters and the deep seafloor

**DOI:** 10.1002/ecy.3898

**Published:** 2023-01-05

**Authors:** Erik Simon‐Lledó, Brian J. Bett, Noëlie M. A. Benoist, Henk‐Jan Hoving, Dmitry Aleynik, Tammy Horton, Daniel O. B. Jones

**Affiliations:** ^1^ National Oceanography Centre Southampton UK; ^2^ GEOMAR Helmholtz Centre for Ocean Research Kiel Kiel Germany; ^3^ Scottish Association for Marine Science Oban UK

**Keywords:** abyss, benthic community, benthic–pelagic coupling, carbon pump, carcasses, Clarion Clipperton zone, food fall, megafauna, micronekton, ocean circulation, *Pleuroncodes*

Massive swarms of the red crab *Pleuroncodes planipes* (Stimpson, 1860), a species of squat lobster, are a dominant functional component of the upwelling ecosystem in the eastern Pacific Ocean (Boyd, 1967; Smith et al., 1975). These swarms can wash ashore on the coast, creating mass depositions of crustacean carcasses, a striking phenomenon that has been long documented in Baja California and California (Aurioles‐Gamboa et al., 1994; Boyd, 1967). However, little is known about the fate of crab swarms transported offshore by oceanic currents. In May 2015, using an autonomous deep‐sea robot, we discovered an unexpectedly large fall of red crab carcasses (>1000 carcasses ha^−1^) at a depth of 4050 m on the abyssal Pacific seafloor (Figure 1), almost 1500 km from their spawning areas off the northwest American coast. Several questions arise from this unexpected finding that may help unveil additional close linkages in nutritional transport between processes at the sea surface and the remote abyssal seafloor.

**FIGURE 1:**
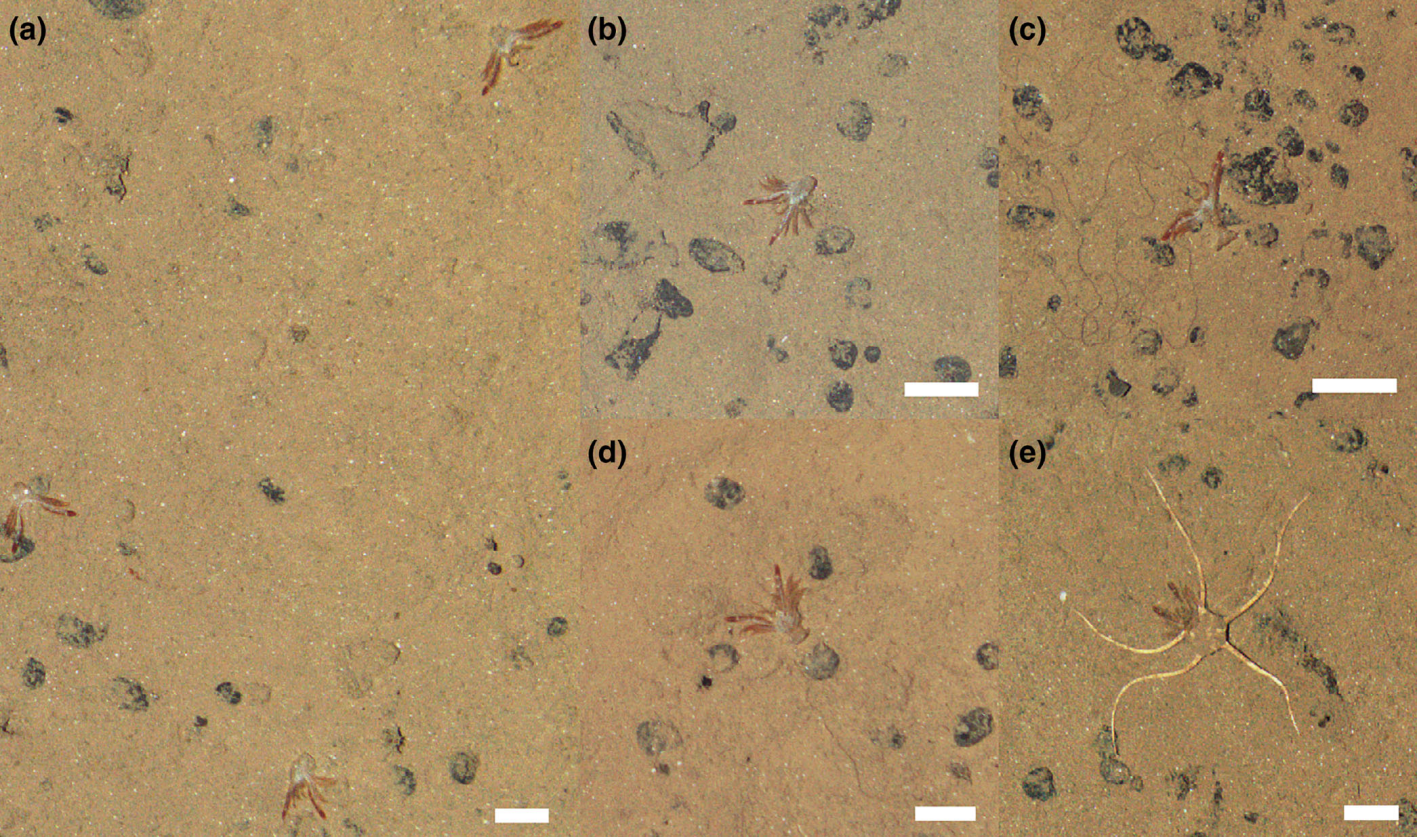
Carcasses of red crab, *Pleuroncodes planipes*, photographed using the autonomous vehicle *Autosub6000* during seabed surveys conducted in the abyssal Northeast Pacific (APEI‐6 site, Clarion Clipperton Zone). (a–e) All images were collected on 7 May 2015 on an abyssal hilltop, at water depths between 4000 and 4050 m within a survey area extending from 17.325° N–122.871° W to 17.279° N–122.900° W. Note the ophiuroid that appears to be feeding on a carcass in (e). Scale bars, 50 mm. Image credit: National Oceanography Centre.

While carrying out an extensive visual survey of the abyssal seafloor in the Pacific, we were surprised by the high abundance of squat lobsters. These crustaceans can be encountered from the poles to the tropics and from intertidal rockpools to the greatest depths of the ocean. However, in the abyss, 4000 m deep, it would be very unusual for them to be the most abundant organism observed. On closer inspection, it became clear that our squat lobsters were all dead, often lying on their back with their abdomen extended, a very unnatural pose for a living squat lobster (Figure 1). Further research suggested we were dealing with a mass fall of *P. planipes*. Carcasses exhibited a surprisingly low state of decomposition, still brilliant red colored in all of the almost intact appendages, though fading in the abdomen. Relatively rapid sinking rates and the large numbers falling might have minimized the scavenging of carcasses during their descent through the water column, as occurs in other massive deep‐sea food falls, like those in jellyfish or pyrosomes (Lebrato et al., 2012; Lebrato & Jones, 2009). In turn, the lack of many scavengers feeding on the carcasses suggested that the deposition was recent, or even ongoing. The densest carcass aggregations were found on an abyssal hilltop (mean density: 1053 carcasses ha^−1^) extending over an area of approximately 30 km^2^. Less dense aggregations (18–240 carcasses ha^−1^) were found in equivalently large seascapes surveyed nearby (a plain and a trough; Figure 2b). Carcass density was relatively variable at fine scales (i.e., a few meters), with patches containing up to three to four carcasses m^−2^ observed mostly in the hilltop area. This finding emphasizes the value of using observations from autonomous underwater vehicles (or other imaging platforms) to supplement and expand physical sample collection methods, for example, trawl, box, and multicore samples collected within the seabed locations imaged (during the same expedition; Jones et al., 2021) missed the carcass fall.

**FIGURE 2:**
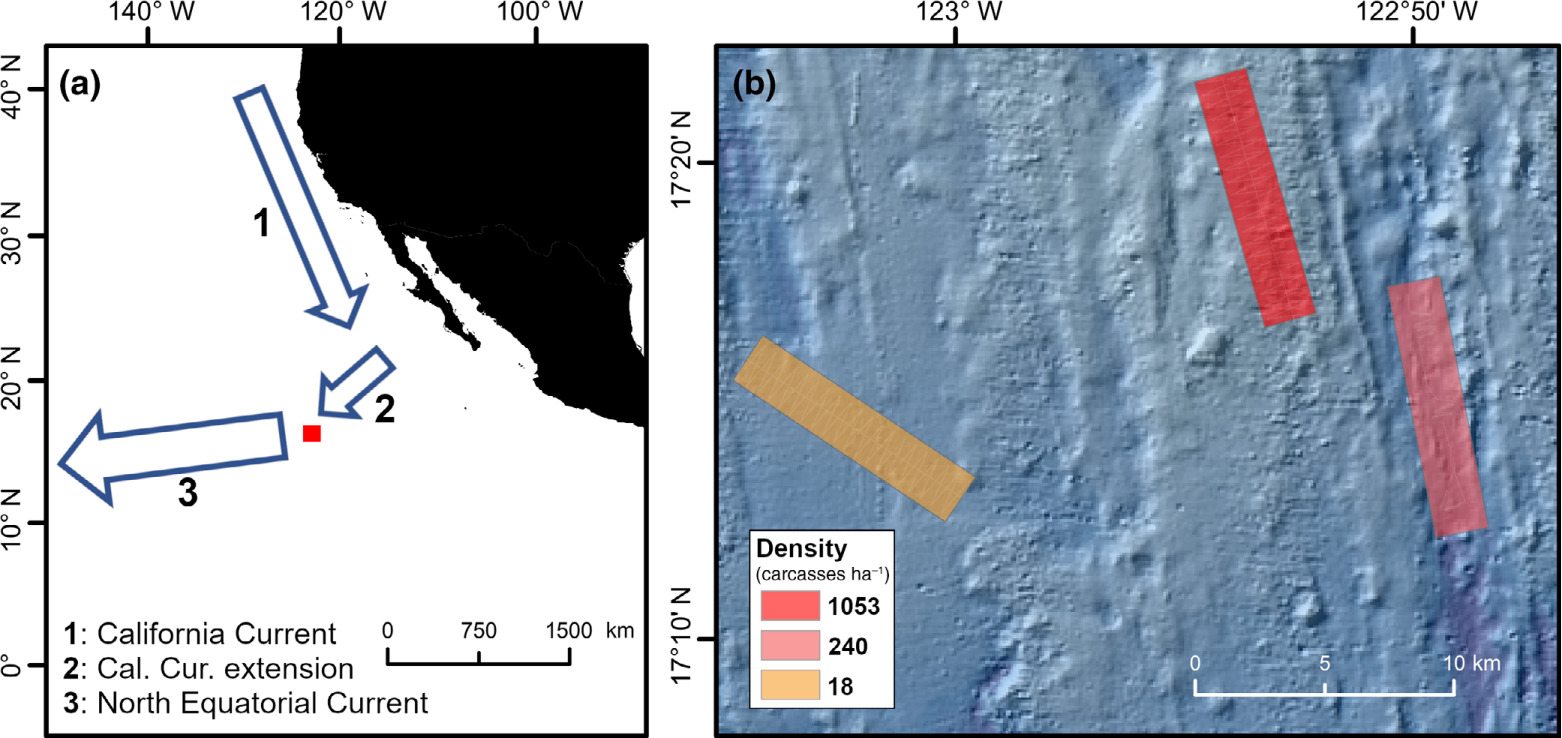
(a) Study area location and (b) different seascapes investigated during *JC120* expedition (Jones et al., 2021) to APEI‐6 site in the Northeast (NE) Pacific. (a) Main superficial water mass flows in NE Pacific Ocean, adapted from Boyd (1967) and Kawabe and Fujio (2010). Red rectangle indicates position of study area. *Cal Cur*: California Current. (b) Mean densities (in carcasses ha^−1^) of dead *Pleuroncodes planipes* encountered on the seabed of each of the three areas surveyed (from left to right: plain, hill, and trough). A total of four (2‐km‐long) seabed transects selected at random were assessed within each seascape. Density ranges: 0–59 carcasses ha^−1^ (plain); 101–3068 carcasses ha^−1^ (hill); and 0–102 carcasses ha^−1^ (trough).

The red crab *P. planipes* is the most abundant species in the micronekton community (size: 20–200 mm) along the southern part of the California Current (i.e., Appendix S1: Figure S2), one of the four major upwelling systems of the world (Robinson et al., 2004). *P. planipes* plays an important role in the cycling of carbon from primary production to higher up into the food chain (Longhurst et al., 1967). Being an important primary consumer and common prey of many marine vertebrates, red crabs are an energetically important link in the food web with a similar trophic role as anchovy and sardine (Robinson et al., 2004; Smith et al., 1975). The larvae, juveniles, and young adults of *P. planipes* are mostly planktonic. Red crabs start the benthopelagic stage of their life cycle, including diurnal migrations, toward the second year of life when their standard carapace length (SCL) is between 17 and 20 mm, until they reach 32–34 mm and become fully benthic (Boyd, 1967). We measured the SCL of a random subset of 800 carcasses identified in scaled seabed imagery. These ranged from 10.5 to 34.2 mm (median length: 21.2 mm; Appendix S1: Figure S1), suggesting all red crabs were planktonic subadults.

Although benthic adult populations are typically found in subtropical continental margins and nearby seamounts at depths of 50–400 m, they have been sighted as far north as Oregon (Sanford et al., 2019) and as far south as Panama (Pineda et al., 2016). The other species in the genus, *P. monodon*, is very similar in both appearance and ecological role (Gutiérrez et al., 2008) but has a more southerly distribution, tending to occur off Chile, and has never been recorded at the latitude of our observations.

Upwelling‐induced phytoplankton blooms attract large aggregations of young adult planktonic red crabs in surface waters (Robinson et al., 2004). These swarms can at times find themselves in disadvantageous oceanographic situations. Crustacean swarms can be washed ashore (Appendix S1: Figure S3), or currents can transport them offshore (southwest) toward a point of no return within the California Current, where they become expatriates that no longer contribute to the maintenance of the species (Longhurst et al., 1967). The fate of the latter we document here.

Our observation revealed a remarkably large aggregation of carcasses at abyssal depths and at a large distance (~1500 km) from their closest known spawning areas. Indeed, the original description of *P. planipes* was made from material collected over 1500 km offshore and 1000 km northwest from our site (Stimpson, 1860), and there is likely an oceanic pathway connecting the spawning areas to the locations of the mass falls. The California Current sweeps surface waters south along the American coast, swinging westward approximately at the latitude of southern Baja California (Boyd, 1967) to ultimately connect with the North Pacific Equatorial Current, flowing westward between 8° and 18° N across the Pacific basin (Kawabe & Fujio, 2010), right above our study area (Figure 2a). However, why so little decomposition was found in crab carcasses seems unclear.

Self‐propagating westward mesoscale eddies are a potentially significant regional mechanism for the material transport of upper seawater layers and its inhabitants thousands of kilometers offshore. These originate under strong winds, blowing through two main gaps in the Sierra Madre Mountains and over the Gulf of Papagayo and Tehuantepec, which are occasionally capable of amplifying abyssal currents in the northeast (NE) Pacific (Aleynik et al., 2017). Averaged over 24 years of satellite observations, the estimates of the radius of relatively stable anticyclonic (rotating clockwise) eddies are 92 km (range 60–110 km), and the average translation speed is 12.5 cm s^−1^ (range 3.4–18.1 cm s^−1^; Purkiani et al., 2020). Therefore, a year is a realistic minimal arrival timescale from the California and Baja California shelf to our site. But a year seems a rather long time for the transport to occur with such little decay, hence other processes might be involved.

Because of the high carcass numbers observed in the abyssal area, we assessed the potential importance of such a large food fall to this typically low‐food environment (e.g., Smith et al., 2008). We did so by estimating the carbon contribution of the food fall based on allometric relationships of the species (i.e., SCL to wet weight; Boyd, 1962) and wet weight to organic carbon conversion factors (Childress & Nygaard, 1974). Based on this analysis, each carcass would contain an average of 390 mg of C_org_ (± 239 mg SD) when fresh. Assuming that the decomposition in the water column appeared to be low and that at least 75% of the carbon was preserved during the abyssal descent, the carbon flux associated with the mass fall over the entire mapped hilltop area (where the fall was most severe) would be equivalent to 0.03 g C_org_ m^−2^. At fine scales, in areas with the highest carcass density, the mass fall could provide as much as 1.23 g C_org_ m^−2^, which is almost 1.5 times the expected yearly flux (i.e., 0.85 g C_org_ m^−2^ year^−1^) of particulate organic carbon from the surface in the study location (e.g., Henson et al., 2012; Appendix S1: Section S3), in a single deposition event.

Although it is unclear how regular these crustacean mass falls are in time or the area they cover, our calculations show that even a single deposition might play a much more important role in the biological carbon pump than was previously known in the otherwise extremely oligotrophic abyssal areas. Abyssal ecosystems are known to be strongly modulated by the quantity and quality of detrital food material sinking from the surface ocean (Smith et al., 2008), which makes seabed communities highly sensitive to variations in this flux (Ruhl & Smith, 2004). It appears remarkable that the mean densities of benthic scavenging megafauna (animals >10 mm) found in our study area (i.e., mostly decapods, isopods, and amphipods: 404 ind ha^−1^; Simon‐Lledó et al., 2019) were substantially larger than those typically reported farther away from the potential influence of the California Current, in more southerly locations within the Clarion Clipperton Zone (i.e., 140–170 ind ha^−1^, Amon et al., 2016; Simon‐Lledó et al., 2020). This distinctive benthic community structure (already in place at the time of deposition) in the APEI‐6 site suggests that red crab mass falls might be a periodic yet spatially restricted event in the NE Pacific abyss. But without more information, it is impossible to establish how often and how widespread these events are in this region or their precise role in deep‐sea food webs.

The discovery of this red crab mass fall and its potential oceanographical, trophic, and ecological implications suggests that the connection between abyssal and surface processes might be tighter than commonly perceived. As with *P. planipes*, other crustacean species also aggregate in large swarms. These include the “langostilla”, *Munida gregaria*, in the western South Pacific (Zeldis & Jillett, 1982), the portunid crab, *Charybdis smithii*, in the Arabian Sea (Christiansen & Boetius, 2000), or krill, *Euphausia superba* in the Southern Ocean (Atkinson et al., 2008) and *Meganyctiphanes norvegica* in the North Atlantic. The latter species are also known to provide food subsidies to deep seabed communities (Christiansen & Boetius, 2000; Hirai & Jones, 2012; Schmidt et al., 2011), and our observations suggest a globally relevant role of crustacean carcasses in deep‐water benthic systems (Halfter et al., 2021). This may not just have ecological implications. Crustacean species like *P. planipes* are a known vector for microplastics (Choy et al., 2019), and mass depositions could facilitate the transport of contaminants from coastal environments to the deep sea. With such a wealth of open questions, dedicated research focusing on the tracking and monitoring of crustacean mass deposition events appears urgent to better understand the periodicity, magnitude, causes, and consequences of these processes in the deep sea.

## AUTHOR CONTRIBUTIONS

Erik Simon‐Lledó, Brian J. Bett, and Daniel O. B. Jones collected the data and conceived the study. Erik Simon‐Lledó and Noëlie M. A. Benoist processed and analyzed image data. Tammy Horton, Henk‐Jan Hoving, and Dmitry Aleynik provided expert insight in data interpretation. Erik Simon‐Lledó composed the manuscript with significant input from all coauthors.

## CONFLICT OF INTEREST

All authors declare no conflicts of interest.

## Supporting information


Appendix S1

## Data Availability

Survey data (Simon‐Lledó et al., 2022) are available in Zenodo at https://doi.org/10.5281/zenodo.7042090.
