# Supplementary material for: Mass falls of crustacean carcasses link surface waters and the deep seafloor
Source: Ecology. 2023 Jan 5;104(2):e3898. doi: 10.1002/ecy.3898 (PMC10078340; doi:10.1002/ecy.3898)
Supplement: Supplementary file 1 — Appendix S1 [file ECY-104-0-s001.pdf]

Mass falls of crustacean carcasses link surface waters and the deep seafloor

Erik Simon-Lledó[1], Brian J. Bett[1], Noëlie M. A. Benoist[1], Henk-Jan Hoving[2], Dmitry Aleynik[3], Tammy Horton[1], and Daniel O. B. Jones[1]

[1]National Oceanography Centre, Southampton, UK
[2]GEOMAR Helmholtz Centre for Ocean Research Kiel, Kiel, Germany
[3]Scottish Association for Marine Science, Oban, UK

Appendix S1

Section S1

Additional analysis

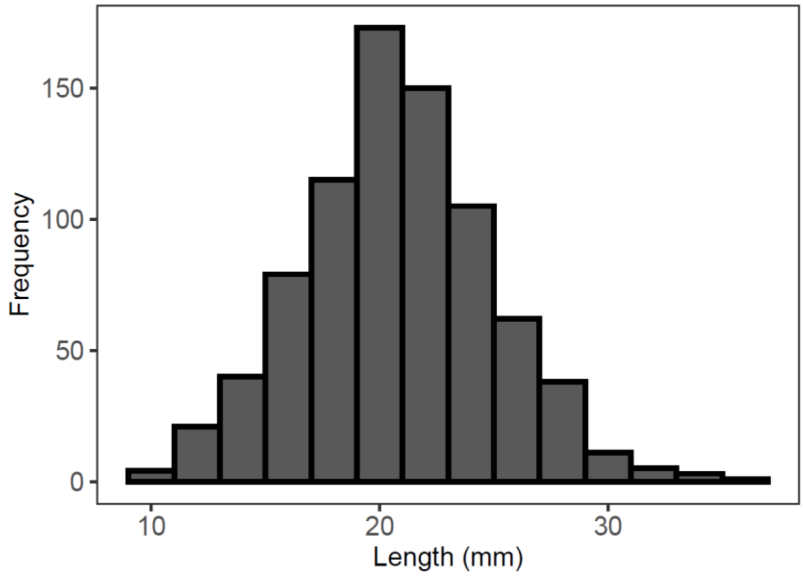

Figure S1. Size-frequency, i.e. Standard Carapace Length (SCL), measured in 800 red crab carcasses encountered 4100 m deep in the abyssal N Pacific seabed (APEI-6 site, Clarion Clipperton Zone)

Section S2

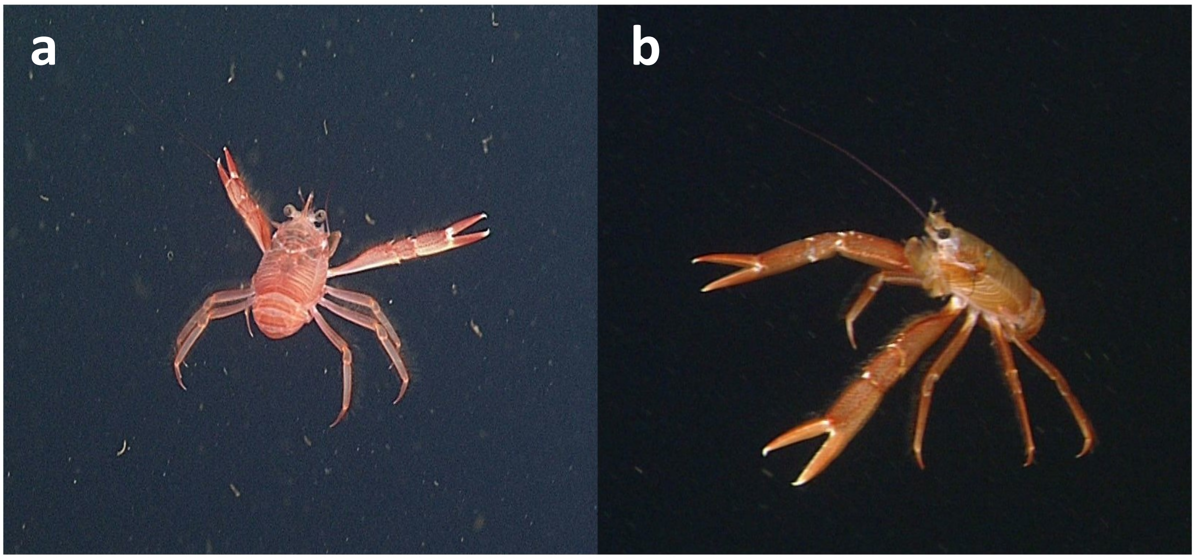

Figure S2. Red crabs, *Pleuroncodes planipes*, photographed at the water column in the eastern north Pacific. **a)** Water depth: 75 m. **b)** Water depth: 102 m. Images courtesy of Monterey Bay Aquarium Research Institute.

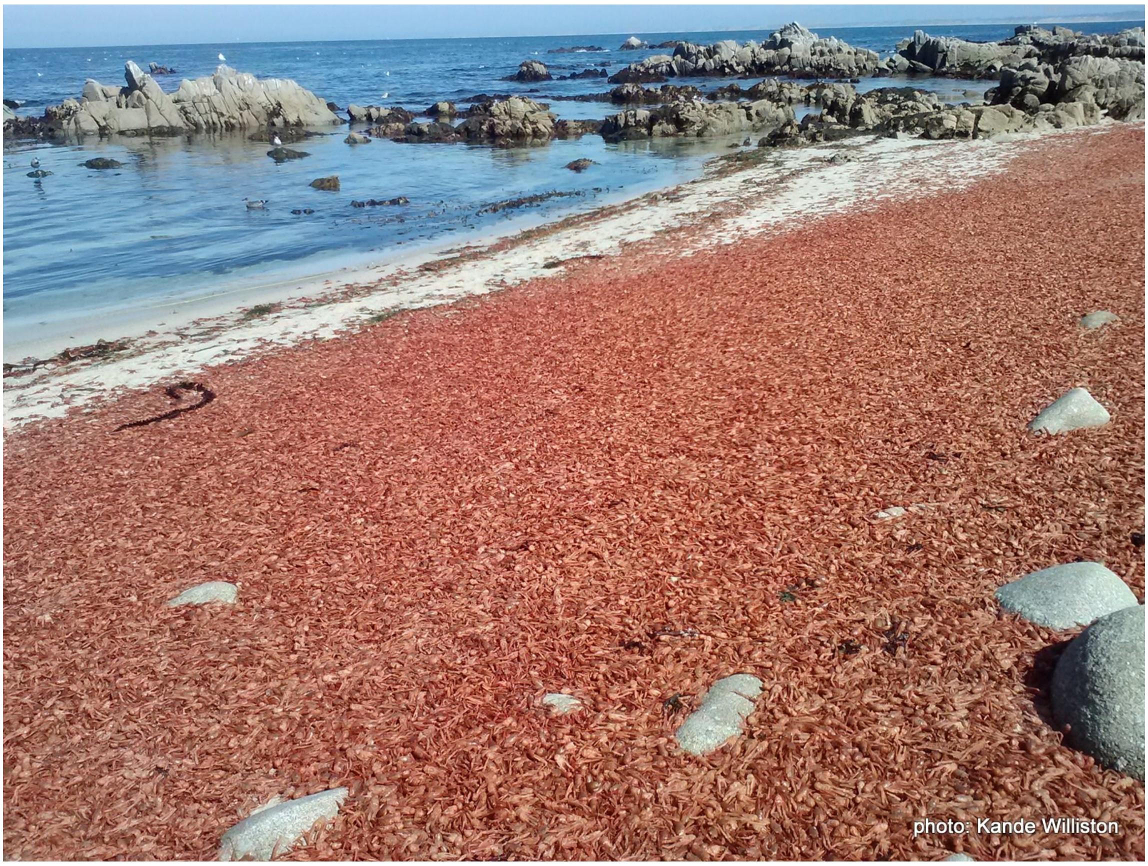

Figure S3. Thousands of red crabs, *Pleuroncodes planipes*, covering Coral Street Beach in Pacific Grove, California (7th October 2015). Image courtesy of NOAA Monterey Bay National Marine Sanctuary; Image credit: Kande Williston.

Section S3

POC flux to the seafloor at study location

Particulate organic carbon (POC) flux to the seafloor across the abyssal N Pacific was estimated by applying a vertical flux attenuation equation to satellite-derived primary production (Carr, 2001) and sea surface temperature based on the relationship defined in Henson et al. (2011). Flux attenuation described by "Martin's b" parameter (Martin et al., 1987) was estimated using a collation of deep moored sediment trap data as described in Henson et al. (2012). Seafloor depth was taken from the ETOP01 global relief model (Amante and Eakins, 2009). All data were spatially averaged to a 1x1 degree grid. POC flux to the seafloor (in g C m^{-2} yr^{-1}) was extracted at the study location using a nearest neighbour approach.

References

Amante, C. and B.W. Eakins, 2009. ETOPO1 1 Arc-Minute Global Relief Model: Procedures, Data Sources and Analysis. NOAA Technical Memorandum NESDIS NGDC-24. National Geophysical Data Center, NOAA. doi:10.7289/V5C8276M

Carr, M.-E. (2001), Estimation of potential productivity in Eastern Boundary Currents using remote sensing, Deep Sea Res., Part II, 49(1–3), 59–80, doi:10.1016/S0967-0645(01)00094-7

Henson, S., R. Sanders, E. Madsen, P. Morris, F. Le Moigne and G. Quartly (2011), A reduced estimate of the strength of the ocean's biological carbon pump, Geophysical Research Letters, 38, L04606, doi:10.1029/2011GL046735

Henson, S., R. Sanders and E. Madsen (2012), Global patterns in efficiency of particulate organic carbon export and transfer to the deep ocean, Global Biogeochemical Cycles, 26, GB1028, doi:10.1029/2011GB004099

Martin, J. H., G. A. Knauer, D. M. Karl, and W. W. Broenkow (1987), VERTEX: Carbon cycling in the Northeast Pacific, Deep Sea Res., Part A, 34(2), 267–285, doi:10.1016/0198-0149(87)90086-0
